# Predictive modelling and ranking: *Azadirachta indica* compounds through indices and multi-criteria decision-making techniques

**DOI:** 10.3389/fchem.2025.1580267

**Published:** 2025-04-29

**Authors:** D. S. Anuradha, B. Jaganathan

**Affiliations:** Department of Mathematics, Vellore Institute of Technology, Chennai, India

**Keywords:** *Azadirachta indica*, neem, topological indices, regression models, QSPR, multi-criteria decision making, VIKOR, SAW

## Abstract

**Introduction:**

*Azadirachta indica* (neem) shows medicinal potential against chronic diseases, but clinical translation is challenging. This study aimed to analyze neem compounds using topological indices (TIs) to predict physicochemical properties.

**Methods:**

Valency-based indices, including Zagreb and atom bond connectivity indices, were used to characterize boiling point, vaporization, enthalpy, mass, and refractivity. Regression analysis and multi-criteria decision-making methods were employed for predictive modeling and compound ranking.

**Results:**

Statistical metrics demonstrated the predictive power of the models. Ranking methods provided a hierarchical ordering of compounds based on therapeutic potential.

**Discussion:**

This study contributes to analogous prediction, optimization, and virtual screening of neem compounds using a cost-effective approach. The findings offer insight into neem compound properties, potentially accelerating drug discovery and development.

## 1 Introduction

*Azadirachta indica*, informally known as neem, is a tree belonging to the family Meliaceae. It is used in pharmaceuticals, animal nutrition, agriculture, cosmetics, dental hygiene, personal care, and fuel production (Nwanekezie et al., 2023). Neem extracts (NEs) have been patented for their antibacterial properties, targeting drug-resistant bacteria, and treating breast carcinomas (Nandi and Ghosh, 2023). NEs have anticancer effects on the liver and lung carcinomas, and clinical trials have shown their safe modulation of biological systems in cancer treatment. Studies have revealed that nimbins, a bioactive neem compound, act against four dengue virus strains (Khan et al., 2024). Continued neem consumption can reduce dengue-related morbidity and human pathogens mortality (Wylie and Merrell, 2022). Research suggests that incorporating neem into anti-diabetic agents enhances their effectiveness (Abdullah et al., 2023). NEs inhibit SARS-CoV-2 3-Chymotrypsin (3C) and impede their adhesion to the vascular epithelium. These studies highlight potential of NE in treating COVID-19 (Johnson et al., 2021). Neem leaf extracts show promise against HIV, malaria, and cancer cell proliferation (Eze et al., 2022). Nimbolide, azadirachtin, and gedunin are bioactive neem compounds that influence biological processes in animals (Sarkar et al., 2021). NEs may be suitable for managing HIV, cancer, skin diseases like psoriasis and obesity (Maji and Modak, 2021; Mohan et al., 2023; Tiwari, 2023). NE’s bactericidal, fungicidal, and insecticidal traits are vital for ecological cultivation with arthropod control (Modi and Soni, 2023). Recent research has focused on exploring the potential of neem as a novel therapeutic agent, particularly for its antimicrobial, anti-inflammatory, antioxidant, and anticancer properties (Puttongsiri et al., 2025). There is growing interest in neem compounds for the treatment of drug-resistant pathogens and biofilm-forming organisms (Kumar Singh et al., 2025).

Although the benefits of *Azadirachta indica* are vast and hold significant pharmacological promise, several challenges hinder its wider clinical application. Various obstacles that impede the advancement of neem-based pharmaceuticals include a) biodiversity, b) toxicity, c) regulatory inconsistencies, d) inefficient and labor-intensive extraction processes, and e) the requirement for precise elucidation of the molecular mechanism (Tembe-Fokunang et al., 2019). A bibliometric analysis of reputable publications and biodiversity studies from various countries concluded that sufficient funding is required for neem research to understand neem biodiversity and toxicity for its safe application in the treatment of diseases (Onasanya et al., 2022). A brief description of these obstacles is as follows:

Being a native to the Indian subcontinent, the neem tree has been successfully introduced to African regions, but its global distribution remains limited as shown in Supplementary Material (Bakewell-Stone, 2024). Research on the toxicity of neem extracts has revealed their effectiveness when administered orally over short periods of time. Nevertheless, prolonged use may result in adverse toxic effects including renal dysfunction, substantial decreases in arterial blood pressure, and hypoglycaemic responses (Braga et al., 2021; Saha et al., 2024; Stinguel et al., 2024; Tumanjong et al., 2024; Ogundipe et al., 2025). Addressing toxicity involves elucidating the precise mechanisms of action, establishing clinical efficacy, and assessing the safety profile of neem-based therapeutics (Modi and Soni, 2023). Furthermore, the development of standardized protocols for extract preparation is essential, because suboptimal processing techniques may lead to detrimental health consequences (Islas et al., 2020).

Internationally recognized databases reveal a significant gap in the information pertaining to the physicochemical characteristics of neem phytochemicals. The phytochemicals in the neem are likely to be composed of numerous flexible molecules. Traditional methodologies struggle to capture the conformation-dependent properties of these molecules and their interactions with biological targets, necessitating interdisciplinary approaches (Ramadhan et al., 2023). The main challenges include: 1) collecting comprehensive, high-quality data on a diverse array of neem compounds, 2) choosing appropriate molecular descriptors, and 3) ensuring rigorous validation and defining the applicability domain of the models. Addressing these challenges is crucial to improve the efficiency and sustainability of neem-derived drug development for chronic diseases (Parmar et al., 2025).

Developing precise QSPR models and prioritizing lead compounds can help to overcome these challenges. These processes facilitate the prediction of properties and hierarchical arrangements that are crucial for efficient virtual screening (Noviandy et al., 2025). QSPR is a theoretical framework used in medical research to enhance the scrutiny of pharmaceutical agents intended to treat specific ailments with desired characteristics derived from their physicochemical characteristics and biological efficacy (Sorgun and Birgin, 2025). This vital methodology supplements experimental research with computational analyses and plays a crucial role in toxicity analysis and virtual screening of pharmaceutical compounds (Hasani et al., 2025).

Molecular descriptors/topological indices are indispensable tools for QSPR studies, enabling the theoretical prediction of physicochemical properties (Huang et al., 2024; Ozge, 2024; Yu et al., 2024). These descriptors accurately identify the information embedded in molecular structures, which is governed by the interconnectivity and spatial configuration of atoms (Sahu and Ojha, 2023). The importance of topological indices (TIs) stems from their ability to facilitate the prediction of their theoretical chemical properties, thereby bolstering QSPR methodologies (Ullah et al., 2023). These numerical descriptors assign a polynomial or number to the correlation between the positions of the atoms in a chemical graph structure and their innate physical properties (Hakeem et al., 2023; Tharmalingam et al., 2023; Yang et al., 2023).

In the literature, the first and second Zagreb indices have applications in complexity and molecular chirality studies of chemical compounds (Hakami et al., 2025). When studying the heat of formation of octanes and heptane, the augmented Zagreb index was found to be a suitable predictive index (Ali et al., 2021). The heat of formation, stability, and strain energy of alkanes and cycloalkanes are highly correlated with the atom bond connectivity (ABC) index (Rahul et al., 2022). Vukičević and Gašperov defined the Adriatic indices. There are three types of indices: variable, discrete, and extended. Discrete Adriatic descriptors are among the closest groups of these descriptors and contain 148 descriptors (Kulli et al., 2021). These indices predict the enthalpy of vaporization, heat capacity, Log P, relative retention time, and biological activity, which are necessary for virtual screening of drug compounds (Anuradha et al., 2024).

TIs have been used in QSPR studies to investigate medications used for diverse medical conditions. The physical attributes of biochemical networks (Ullah et al., 2024), antiviral drugs used for treating headaches (Sardar et al., 2023), and nonsteroidal anti-inflammatory drugs, including opiates and antidepressants, (Gnanaraj et al., 2023), were analyzed by TIs. Drugs used for cardiac dysfunction (Kuriachan and Parthiban, 2025), schizophrenia, tuberculosis, malignancies, viral infections, cancer (Arockiaraj et al., 2025; Kuriachan and Angamuthu, 2025; Nasir, 2025; Qin et al., 2025; Sorgun and Birgin, 2025), and respiratory disorders, such as asthma, were analysed by TIs (Adnan et al., 2022; Balasubramaniyan and Chidambaram, 2023; Gnanaraj et al., 2023; Hakeem, 2023; Huang et al., 2023; Pattabiraman and Cancan, 2023; Zaman et al., 2023). The properties crucial to COVID-19 medicines and anti-hepatitis drugs were investigated using M-polynomial and NM-polynomial (Asghar, 2025), geometric-quadratic index, quadratic-geometric indices, and the first and second inverse Nirmala indices (Das et al., 2023; Nagarajan et al., 2023). Adriatic indices have been used to analyze curcumin- and benzophenone-conjugated PAMAM dendrimers, boron triangular nanosheets, and some nanostructures (Anuradha and Jaganthan, 2023; Anuradha and Jaganathan, 2023).

Multiple-criteria decision-making (MCDM) methodologies, such as VIseKriterijumska Optimizacija I Kompromisno Raspoređivanje (VIKOR) and Simple Additive Weighting (SAW), enable hierarchical ordering of phytochemicals based on various parameters (Zuo et al., 2023). Hierarchical ordering in drug design streamlines discovery by prioritizing candidates based on criteria. This method optimizes resources, provides structure-activity relationship insights, facilitates data-driven decisions, and optimizes combinatorial chemistry. By ranking compounds, researchers identify promising candidates, allocate resources effectively, navigate large libraries and increase success in developing new therapeutics. Various drugs used for treating lung disorders (Ashraf and Idrees, 2024), cancer (Li et al., 2022; Farooq, 2024), eye disorders, kidney cancer (Husin et al., 2024), anti-psychotic drugs (Saeed and Idrees, 2024),and multiple sclerosis (Farooq et al., 2025) have used MCDM to rank drug compounds.

QSAR/QSPR analysis of neem compounds has traditionally been conducted using experimental methods. Recent investigations have utilized laboratory techniques, such as gas chromatography-mass spectrometry and liquid chromatography coupled with quadrupole time-of-flight mass spectrometry, to identify and quantify the bioactive constituents in neem extracts. Density Functional Theory analysis remains the sole computational method employed to evaluate the reactivity and stability of neem phytochemicals (Costa et al., 2021; Ojha et al., 2021). Neem compounds present substantial opportunities for drug discovery and optimization; however, many of their critical properties have not been sufficiently investigated.

In literature, phytochemical compounds like curcumin, resveratrol were examined widely using TIs (Çolakoǧlu, 2022; Preetha et al., 2024; Zaman et al., 2024). However, neem compounds’ pharmacological activities through molecular descriptors remains unexplored. Hence this article attempts to explore them through QSPR modelling and ranking techniques. Linear and quadratic regression methods were applied to create QSPR models. The model was developed using a set of descriptor formulations linked to specific physicochemical properties of a range of chemicals that exhibited biological activity. The results were used to evaluate and rank the neem chemicals. The implications of these findings can enhance pharmacist capabilities across various stages of drug development, namely, analogs evaluation, predicting drug compositions, and virtual screening.

## 2 Materials and methods

### 2.1 Neem phytochemicals

Neem, the botanical entity known as a “Pharmaceutical Wonder,” is a repository for an extensive array of medicinal properties. This plant contains approximately 300 distinct phytochemicals, each characterized by its unique chemical composition and structural complexity. The therapeutic efficacy of neem is attributed to its complex phytochemical profile, which includes gallic acid, limonoids, saponins, nimbins, catechins, glycoproteins, and flavonoids, which contribute to its diverse medicinal properties (Sandhir et al., 2021). This study focused on a subset of 11 phytochemicals selected for their extensive research history and frequent utilization across various scientific disciplines, namely, azadirone, nimbin, nimbolide (Sarkar et al., 2021), azadirachtin (Nagini et al., 2024), stigmasterol (Bakrim et al., 2022), tiglic acid (Khanpara and Jadeja, 2022), catechin (Monika et al., 2023), scopoletin (Antika et al., 2022), odoratone, tirucallol (Fernandes et al., 2019), and sugiol (Bajpai et al., 2021). The structures of the phytochemicals (as per PubChem database) are shown in Table 1.

**TABLE 1:** Therapeutic neem phytochemicals - molecular structure.

Structure of potent Azadrichita indica bio active compounds	
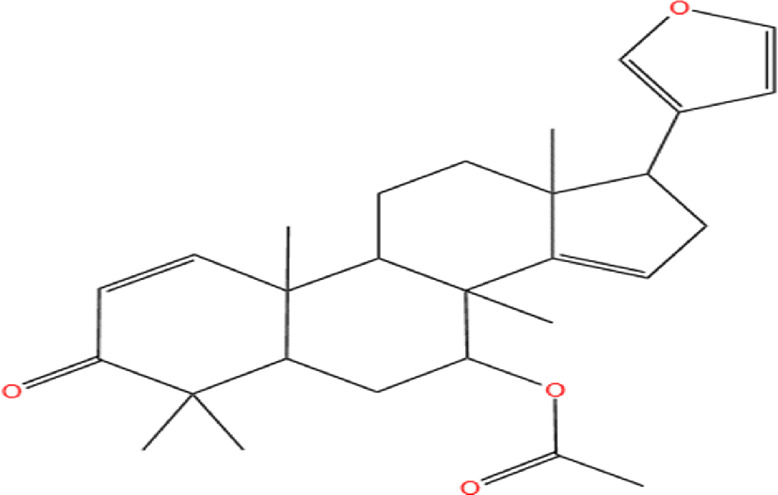	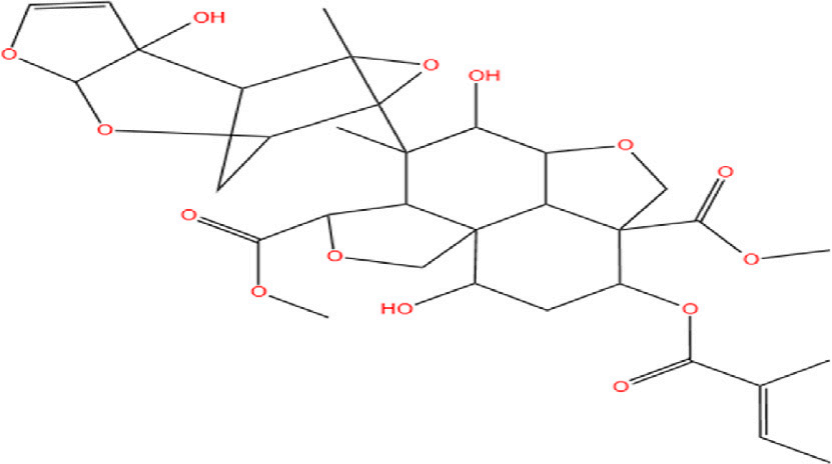
(a) Azadirone	(b) Azadirachtin
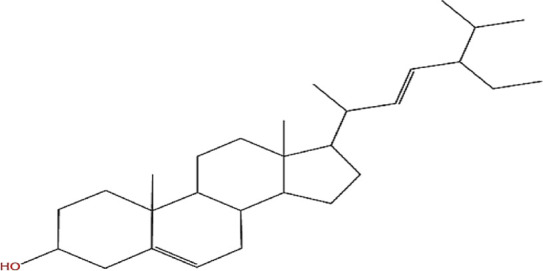	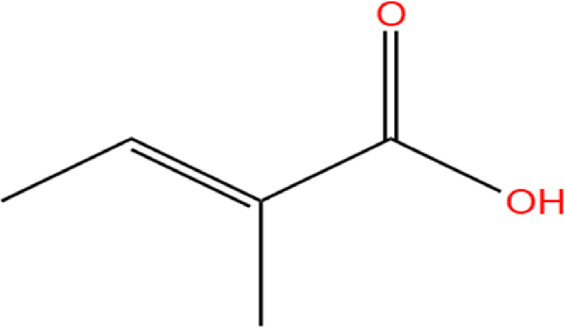
(c) Stigmasterol	(d) Tiglic Acid
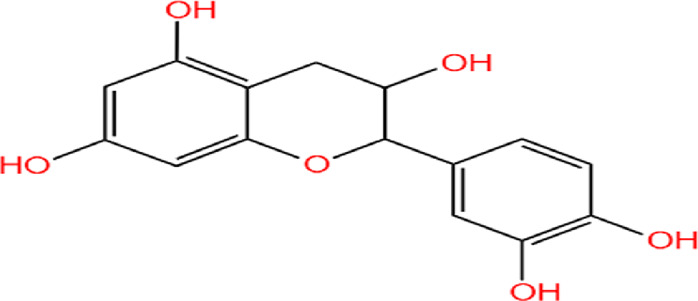	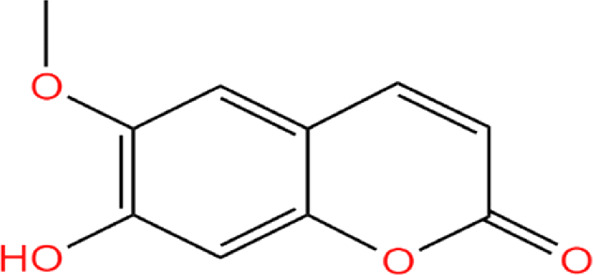
(e) Catechin	(f) Scopoletin
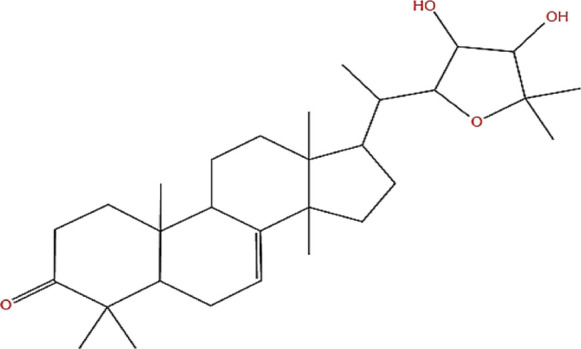	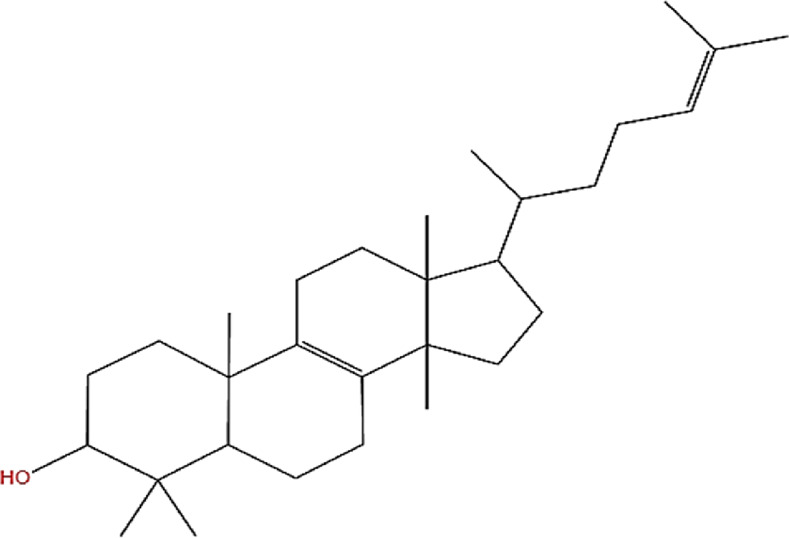
(g) Odoratone	(h) Tirucallol
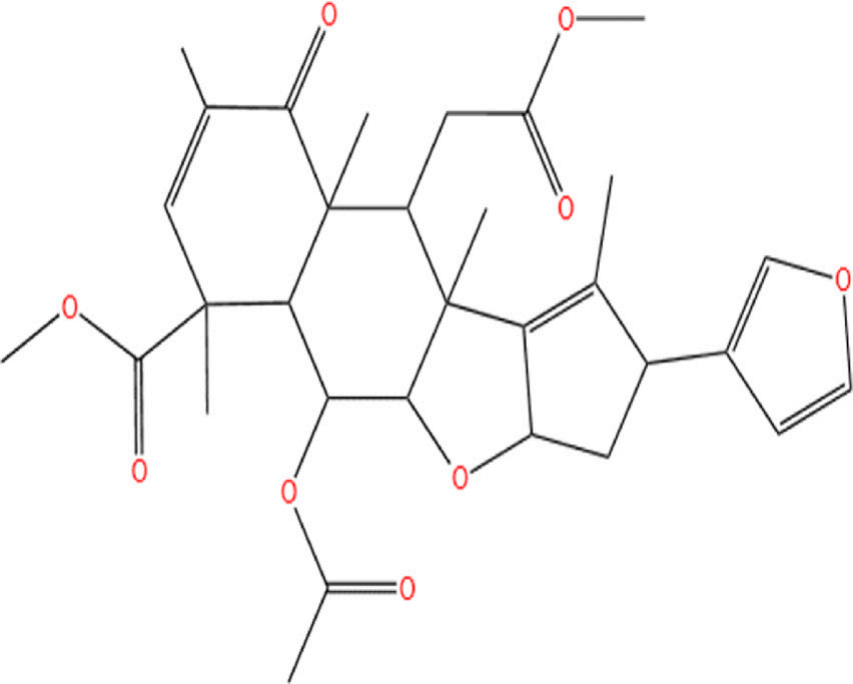	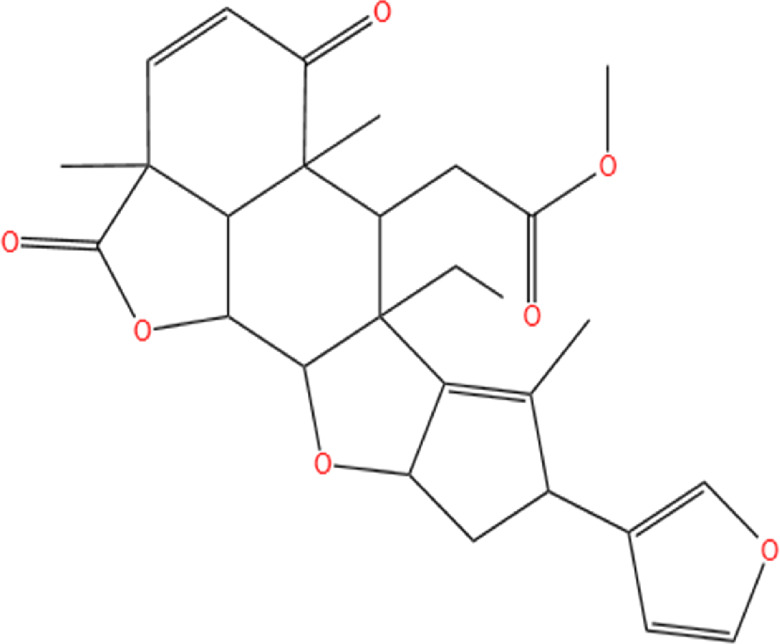
(i) Nimbin	(j) Nimbolide
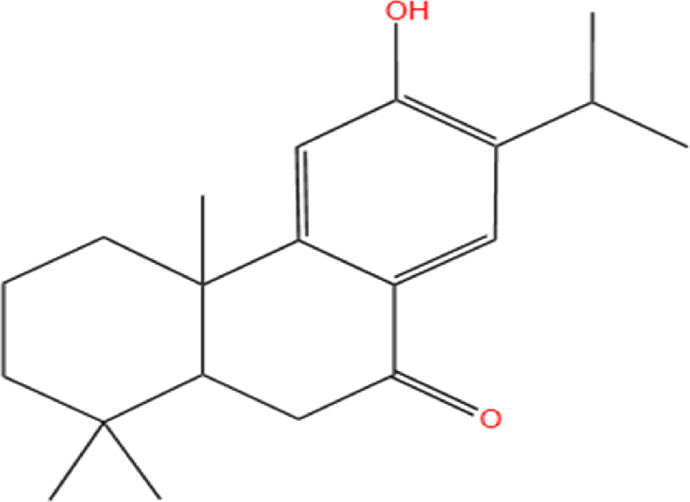	
(k) Sugiol	

### 2.2 Valency based indices and drug likeness prediction

Numerous physicochemical characteristics are crucial for predicting drug-likeness, particularly Absorption, Digestion, Metabolism, Excretion, and Toxicity (ADMET) properties, which are governed by Lipinski’s rule of five. However, these properties of neem phytochemicals have not been extensively studied, leading to a scarcity of data in internationally recognized databases. TI is a crucial alternative method for analyzing and predicting the properties of these compounds. This study employed valency-based topological indices (TIs) to describe a range of chemical, physical, and biological activities using edge partition technique. The following section offers a succinct overview of the properties and indices expected to predict the properties examined in this study (Abdullah et al., 2023).

The efficacy of pharmaceutical compounds is significantly influenced by their solubility, with more soluble compounds generally demonstrating a higher potency. The solubility is determined by the octanol-water partition coefficient, expressed as Log P, which can be derived from the boiling point of a molecule (Karami et al., 2022). The dissolution process is affected by polarizability and vaporization enthalpy, which involves heat absorption. These characteristics are linked to enhanced drug effects and mitigated impact reduction due to evaporation. In pharmaceutical research, mass determination is critical because it influences whether substances remain suspended or sink in the liquid media. For pharmacological molecules, a lower mass is typically preferred because of its association with crystallization processes. Drug behavior is also influenced by molar refraction, a property that is related to both refractive index and polarizability. Increased refractive index and polarizability can enhance the interaction of a drug with light and other molecules, making these properties particularly relevant for phototherapeutic applications (Idrees et al., 2025). These attributes play a significant role in drug absorption, distribution, and formulation. This study aims to classify and rank NEs based on their physicochemical properties by deriving the QSPR for vaporization enthalpy, boiling point, polarizability, molar refractivity, and monoisotopic mass (Nagar et al., 2020; Khalikova et al., 2023). TIs, hypothesized to be the best predictors of the aforementioned properties, were used in this study. The following section offers a concise description of the definitions, notations, terminology, and formulations of the TIs utilized in this study.

Throughout this article, Ω denotes any chemical graph, $V\left(\Omega \right)\text{ is}$ the set of all vertices $\left\{\xi ,\xi_{1},\xi_{2},\ldots \xi_{m}\right\},E\left(\Omega \right)$ represents the collection of all edges $\left\{\partial {,\partial }_{1},\partial_{2},\partial_{3}\ldots \partial_{n}\right\}$ . Two vertices ξ ,and $\xi_{1}$ , are said to be neighbors if there exists an edge to link them. Let ∂ represent an edge in the graph $E\left(\Omega \right)$ that ends at ξ and $\xi_{1}$ . The number of edges incident on any vertex ξ is the degree of the vertex and is denoted by $d_{\xi }$ . Degree corresponds to the valency of atom. TIs are based on the edge-partition technique, whose summation runs over all partitions. The indices utilized in this study are formulated in Equations 1–10.

The atom bond connectivity (ABC) index was formulated based on the connectivity between the atoms proposed by Estrada et al., as follows:

$$\sum_{\partial \in E\left(\Omega \right)}\sqrt{\frac{d_{\xi }+d_{\xi_{1}}-2}{d_{\xi }d_{\xi_{1}}}}$$ (1)

The first Zagreb index (M_1_) is one of the elementary indices established by Trinajstic and Gutman and is defined as

$$\sum_{\partial \in E\left(\Omega \right)}d_{\xi }+d_{\xi_{1}}$$ (2)

An augmented Zagreb index (AUZ) was developed based on the ABC index.

$$\sum_{\partial \in E\left(\Omega \right)}{\left[\frac{d_{\xi }\times d_{\xi_{1}}}{d_{\xi }+d_{\xi_{1}}-2}\right]}^{3}$$ (3)

The randic type lodeg index (Heat Capacity predictor) is denoted by RLI and is defined by

$$\sum_{\partial \in E\left(\Omega \right)}\ln\left(d_{\xi }\right)\ln\left(d_{\xi_{1}}\right)$$ (4)

The sum lordeg index (a good predictor of Log P Value) is denoted by SLI and is defined by

$$\sum_{\partial \in E\left(\Omega \right)}\left(\sqrt{\ln\left(d_{\xi }\right)}+\sqrt{\ln\left(d_{\xi_{1}}\right)}\right)$$ (5)

The inverse sum indeg index (a good predictor of TSA) is denoted by ISI and is defined as:

$$\sum_{\partial \in E\left(\Omega \right)}\left(\frac{d_{\xi }d_{\xi_{1}}}{d_{\xi }+d_{\xi_{1}}}\right)$$ (6)

The misbalance lodeg index (a good predictor of Enthalpy of Vaporization) is denoted by MLI and is defined by

$$\sum_{\partial \in E\left(\Omega \right)}\left|\ln\left(d_{\xi }\right)-\ln\left(d_{\xi_{1}}\right)\right|$$ (7)

The misbalance index (a good predictor of the standard enthalpy of vaporization) is denoted by MDI and is formulated as

$$\sum_{\partial \in E\left(\Omega \right)}\left|d_{\xi }-d_{\xi_{1}}\right|$$ (8)

The max-min rodeg index denoted as MMRDI (a good predictor of density), is defined by

$$\sum_{\partial \in E\left(\Omega \right)}\sqrt{\frac{\max\left\{d_{\xi },d_{\xi_{1}}\right\}}{\min\left\{d_{\xi },d_{\xi_{1}}\right\}}}$$ (9)

The inverse sum lordeg index (ISLI), a good predictor of total surface area, is defined by

$$ISLI\left(\Omega \right)=\sum_{\partial \in E\left(\Omega \right)}\left(\frac{d_{\xi }d_{\xi_{1}}}{d_{\xi }{+d}_{\xi_{1}}}\right)$$ (10)

The physicochemical properties taken from Chemspider and calculated indices numerical values are shown in Tables 2, 3 respectively.

**TABLE 2:** Physicochemical characteristics of bioactive compounds from *Azadirachta indica*.

Property	Enthalpy of vaporization	Boiling point	Polarizability	Molar refractivity	Monoisotopic Mass
NEs					
Azadirone	77.6	506	49	123.7	436.26
Azadirachtin	131.3	792.4	66.6	168	720.26
Stigmasterol	88.7	501.1	51.2	129.1	412.37
Tiglic Acid	47.9	198.5	10.6	26.7	100.05
Catechin	—	630.4	29.2	73.6	290.08
Scopoletin	69.2	413.5	19.2	48.3	192.04
Odoratone	98.4	571.2	53.6	135.2	472.36
Tirucalol	88.3	498.9	52.9	133.4	426.39
Nimbin	90.1	606.1	54.8	138.1	540.24
Nimbolide	90.4	608.6	47.7	120.4	466.2
Sugiol	72.1	437.2	35.5	89.7	300.21

**TABLE 3:** Topological indices for analysis of *Azadirachta indica* phytochemicals.

TI	ISI	MDI	MMRDI	ISLI	ABC	M_1_	RLI	SLI	AUZ	MLI
NEs										
Azadirone	43.845	44	47.664	21.547	25.983	198	27.3274	64.193	301.78	18.6562
Azadirachtin	70.2026	68	76.437	35.887	40.875	306	44.4979	99.675	542.53	29.7
Stigmasterol	38.99	40	42.897	20.273	23.67	168	25.6985	50.483	271.2	14.8535
Tiglic Acid	5.7	8	9.4282	4.8263	4.53	26	1.6684	7.9502	37.516	4.7998
Catechin	26.35	23	29.582	11.686	16.647	114	20.7007	39.726	174.44	10.7632
Scopoletin	16.9667	13	16.901	10.646	10.744	72	9.7478	25.705	97.656	6.5392
Odoratone	53.8	54	45.083	24.777	27.692	208	31.1197	58.012	381.57	20.977
Tirucalol	40.6857	42	53.359	17.359	30.342	180	24.5695	59.16	278.09	17.96915
Nimbin	50.547	52	53.283	24.929	29.388	209	32.2078	73.497	350.3	19.0154
Nimbolide	48.5143	40	49.691	22.897	27.275	237	31.4463	70.153	270	17.1059
Sugiol	28.4089	30	33.205	15.01	17.538	118	16.8485	41.18	187.65	13.2349

**FIGURE 1:**
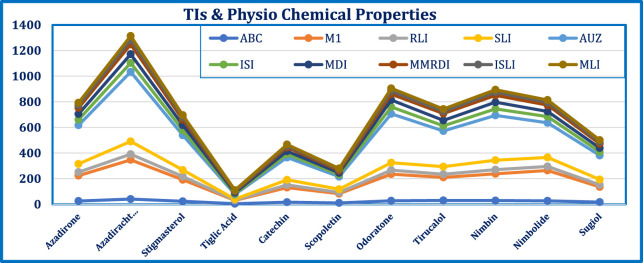
TIs and Properties of Neem compounds - Graphical Representation.

To aid a better understanding graphical representation is provided in Figure 1.

**FIGURE 2:**
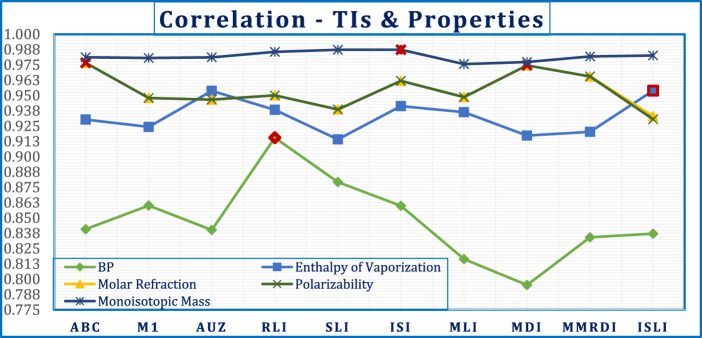
Correlation coefficient between Properties and TIs of Azadrichta Indica.

The correlation coefficient is a crucial metric for evaluating the efficacy of any QSPR model. The computed correlation coefficients are graphically shown in Figure 2.

### 2.3 Regression model for phytochemicals of neem

Linear regression is a widely employed algorithm with a high level of predictability and adaptability, and is easily interpretable for QSPR analysis. The physicochemical properties were viewed as the dependent variables in this model, and the TIs of neem chemicals were considered as the independent variables. The following equations provide linear and quadratic regression models: $PC=A+\left[B\times TI\right]$

$$PC=A+\left[B\times TI\right]+\left[C\times {TI\;}^{2}\right]$$

where PC (dependent variable) denotes the physico chemical property of phytochemical, A is the intercept (constant), B and C denote the regression coefficient (constant), and TI is the topological index (independent variable). The constants A (intercept) and B (regression coefficient) were computed for the five physicochemical properties and ten TIs considered for the study.

### 2.4 Ranking of compounds

The hierarchical arrangement of drug compounds is a crucial component of the virtual screening methodology. Machine learning derived MCDM techniques are pivotal in compound ranking. This study used the VIKOR and SAW rankings to classify neem compounds and evaluate their comparative accuracy. These techiques facilitates drug design by prioritizing candidates, assessing drug-likeness, balancing multiple parameters, and supporting decision making (Magaji Yuguda et al., 2023). This process helps researchers to focus on the most promising compounds with desired properties, such as potency, selectivity, and safety. Ranking allows the rapid screening of large compound libraries by reducing the number of experimental assays required (Gates and Hamed, 2020). The following sections provide a detailed explanation of this process.

#### 2.4.1 VIKOR ranking

VIKOR, an acronym for VIseKriterijumska Optimizacija I Kompromisno Raspoređivanje, represents a sophisticated MCDM approach. This methodology facilitates the selection of an optimal solution by concurrently optimizing multiple parameters. In the pharmaceutical industry, VIKOR is used to coordinate drug candidates and identify a compromise solution that accurately approximates the ideal. This technique is particularly valuable in contexts where a balanced solution is more appropriate than an absolute optimal choice. The emphasis on compromise ranking in VIKOR is especially beneficial when no single pharmaceutical agent satisfies all ideal criteria. It considers both group utility ( S ) and individual regret ( R ) to avoid bias. This methodology provides a clear hierarchical structure and decision-making stability (Gerdes et al., 2021; Feng et al., 2022). The processes involved in this ranking system is described as follows.

##### 2.4.1.1 Alternatives, criterions, and decision matrix

The compounds that need ranking are termed a set of alternatives ( $\alpha_{m}$ ). Indices that influence the drug likeness of compounds are criteria. The indices providing the best-fit regression models derived from QSPR analysis were considered as criteria $\left(\beta_{n}\right.$ ). Construct a decision matrix *γ* $=\left[\theta_{mn}\right]$ , where $\theta_{mn}$ represents the performance of alternatives $\alpha_{m}$ under criterion $\beta_{n}$

##### 2.4.1.2 Criteria weights

To reflect the relative importance of each criterion in the decision process, weights were assigned through a systematic approach, satisfying $\sum_{n=1}^{k}w_{n\;}=1$

##### 2.4.1.3 Best (maximum) and worst (minimum) alternatives

Identify the maximum and minimum of the alternatives.

The beneficial criteria are calculated using ${A_{n}}^{*}=\max_{m}\theta_{mn}\;,$ ${A_{n}}^{-}=\min_{m}\theta_{mn}$

The non-beneficial criteria are calculated using ${A_{n}}^{*}=\min_{m}\theta_{mn}\;,$ ${A_{n}}^{-}=\max_{m}\theta_{mn}$

Utility and Regret Measures: Compute the Utility Measure $S_{i}$ and the Regret Measure $R_{i}$

Utility Measure: $S_{i}=\sum_{n=1}^{k}\left[w_{n}\times \frac{{A_{n}}^{*}-\theta_{mn}}{{A_{n}}^{*}-{A_{n}}^{-}}\right]$ , Regret Measure: $R_{i}=\max\left(w_{n}\;\frac{{A_{n}}^{*}-\theta_{mn}}{{A_{n}}^{*}-{A_{n}}^{-}}\right)$

##### 2.4.1.4 VIKOR index

Parameter λ helps balance the majority rule and individual dominance. Compute the VIKOR Ranking with the VIKOR index $\left(\Omega_{m}\right)$

$$\Omega_{m}=\lambda \;\frac{S_{i}-S^{*}\;}{S^{-}-S^{*}}+\left(1-\lambda \right)\frac{R_{i}-R^{*}\;}{R^{-}-R^{*}}$$

where λ is the weight of the strategy, typically λ=0.5 , where $S^{*}=\min_{i}S_{i}$ , $S^{-}=\max_{i}S_{i}$ , $R^{*}=\min_{i}R_{i}$ , $R^{-}=\max_{i}R_{i}$

##### 2.4.1.5 VIKOR ranking

Arrange alternatives $\Omega_{m}$ in ascending order. The alternative possessing the minimal value is ranked as one (best alternative), and the rest follow in order.

#### 2.4.2 SAW ranking

The Simple Additive Weighting (SAW) method is a widely used multicriteria decision-making approach for ranking alternatives based on multiple criteria and their associated weights. SAW is characterized by their straightforward nature and ease of implementation, rendering them a prevalent choice for decision-making across various domains, including business, finance, and project management (Peng et al., 2021; Feng et al., 2022; Idrees et al., 2025). This process is delineated into distinct phases.

1. Define decision matrix $\theta_{mn}$ using the criterions $\beta_{n}$ used to evaluate the alternatives $\alpha_{m}$

2. Normalize the decision matrix to ensure that all criteria are on the same scale and allows comparison using ${\theta_{mn}}^{*}=\frac{\theta_{mn}}{\sqrt{\sum_{m=1}^{k}{\theta_{mn}}^{2}}}$ . This process ensures that all alternatives are normalized such that one criterion does not dominate because of a larger magnitude.
3. Weights and Weighted Scores: The Weights are assigned to each criterion to reflect their relative importance. In this study, they were determined based on their performance in producing the best-fit regression models satisfying the equation $\sum_{n=1}^{k}w_{n\;}=1$ . The weighted scores $\left(S_{mn}\right)$ are calculated for each alternative on each criterion by multiplying its performance value by the corresponding weight: Weighted Score $\left(S_{mn}\right)=\theta_{mn}\times w_{n}$

4. Aggregation: This total score for each alternative on each criterion is termed as the total score and calculated by: Total Score $\left({TS}_{m}\right)=\sum_{n=1}^{k}S_{mn}$

5. The evaluation of alternatives was conducted through a hierarchical ranking system, where higher numerical values corresponded to enhanced effectiveness.

## 3 Results

A regression-based QSPR model was developed using degree-based TIs derived from the molecular structures. The statistical parameters were characterized as follows. The correlation coefficient (R) was used to quantify the extent of data variance, model fit, and predictive capacity of the relationship. The squared correlation coefficient served as a metric to evaluate the reproducibility of the experimental data (R^2^). The robustness of a model is assessed by statistical metrices. Specifically, a model demonstrates a strong predictive capability when its p-value, calculated from F-statistics, falls below 0.05 and its R-value meets or exceeds 0.6. In this context, N denotes the number of data points in the sample.

### 3.1 Linear regression

**FIGURE 3:**
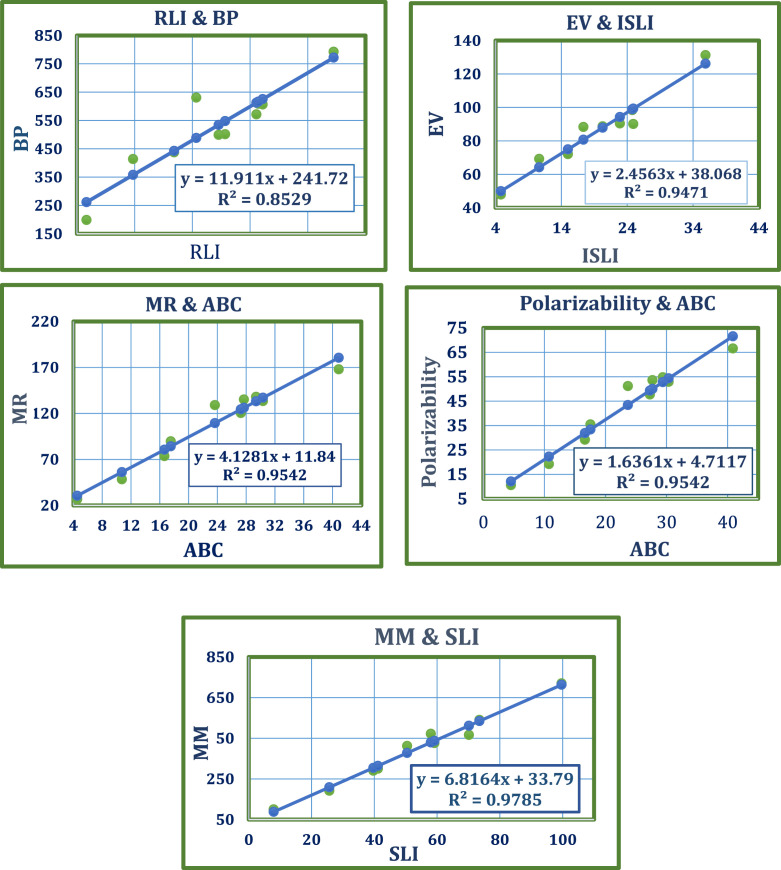
Best fit plots: Linear regression of neem phytochemicals.

Statistical analysis showed all indicators were significant, with p-values below 0.05 and correlation coefficients over 0.78. The metrics confirmed the model’s significance and fit, with the best fit linear regression models and their metrics summarized in Table 4. Figure 3 shows the obtained results.

**TABLE 4:** Statistical Metrics obtained from Linear Regression Analysis.

Property and TI	BP and RLI	Enthalpy of vaporization and ISLI	Molar refraction and ABC	Polarizability and ABC	Monoisotopic Mass and SLI
NEs Statistical Parameter					
N	11	10	11	11	11

R^2^	0.8381	0.9100	0.95382	0.95389	0.9748

F- statistics	46.61	80.96836	185.8923	186.1926	348.299
p	0.000	0.000	0.000	0.000	0.000
A	11.778	2.4211	4.1407	1.641	6.7562
B	239.32	37.426	11.964	4.7596	33.822

### 3.2 Quadratic regression

**FIGURE 4:**
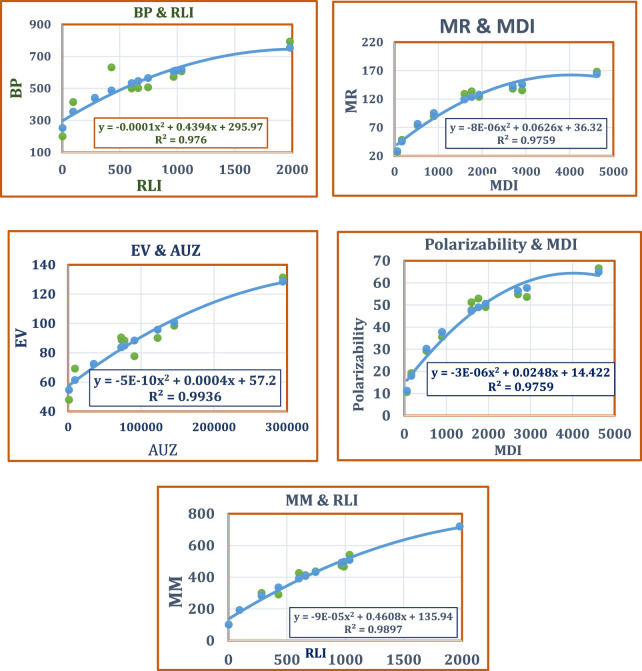
Best Fit Quadratic Regression Models: *Azadirachta indica* compounds.

The statistical parameters listed in Table 5 corroborate the optimal fit and the statistical significance of the quadratic regression model. Visual depictions of the result are in Figure 4.

**TABLE 5:** Statistical Metrics obtained from Quadratic Regression Analysis.

Property and TI	BP and RLI	Enthalpy of vaporization and AUZ	Molar refraction and MDI	Polarizability and MDI	Monoisotopic Mass and RLI
NEs Statistical Parameter					
N	11	10	11	11	11

R^2^	0.83912	0.918,456	0.9779	0.97774	0.97952

F- statistics	20.8633	39.42159	177.3291	175.7358	191.3124
p	0.0006	0.000155	2.37E-07	2.45E-07	1.76E-07
*C*	−0.0001	-5E-10	-8E-06	-3E-06	-9E-05
B	0.4394	0.0004	0.0626	0.0248	0.4608
A	295.97	57.2	36.32	14.422	135.94

### 3.3 VIKOR ranking of neem chemicals

TIs yielding superior regression model fits were used to rank compounds. The relevant data for the alternatives and criteria employed in this ranking procedure are presented in Table 6.

**TABLE 6:** Compounds (alternates) and indices (criterion).

S. No	Compound	ABC	AUZ	RLI	SLI	MDI	ISLI
1	Azadirone	25.983	301.78	27.3274	64.193	44	21.547
2	Azadirachtin	40.875	542.53	44.4979	99.675	68	35.887
3	Stigmasterol	23.67	271.2	25.6985	50.483	40	20.273
4	Tiglic Acid	4.53	37.516	1.6684	7.9502	8	4.8263
5	Catechin	16.647	174.44	20.7007	39.726	23	11.686
6	Scopoletin	10.744	97.656	9.7478	25.705	13	10.646
7	Odoratone	27.692	381.57	31.1197	58.012	54	24.777
8	Tirucalol	30.342	278.09	24.5695	59.16	42	17.359
9	Nimbin	29.388	350.3	32.2078	73.497	52	24.929
10	Nimbolide	27.275	270	31.4463	70.153	40	22.897
11	Sugiol	17.538	187.65	16.8485	41.18	30	15.01

The distribution of criterion weights is predicated on the effectiveness of the TIs in constructing the optimal linear and quadratic regression models. Table 7 delineates the allocated weights, which are collectively summed to unity.

**TABLE 7:** Assigned criterion weights.

Index	ABC	AUZ	RLI	SLI	MDI	ISLI
Weight	0.2	0.1	0.3	0.1	0.2	0.1

The positive and negative ideal solutions of this study are listed in Table 8.

**TABLE 8:** The positive and negative ideal solutions.

Compound	ABC	AUZ	RLI	SLI	MDI	ISLI
Maximum	40.875	542.53	44.4979	99.675	68	35.887
Minimum	4.53	37.516	1.6684	7.9502	8	4.8263

The normalized decision matrix used for the VIKOR is derived from the decision matrix is in Table 9.

**TABLE 9:** Normalised decision matrix of bioactive *Azadirachta indica* compounds for VIKOR.

S. No	Compound	ABC	AUZ	RLI	SLI	MDI	ISLI
1	Azadirone	0.0819	0.0477	0.1203	0.0387	0.0800	0.0462
2	Azadirachtin	0.0000	0.0000	0.0000	0.0000	0.0000	0.0000
3	Stigmasterol	0.0947	0.0537	0.1317	0.0536	0.0933	0.0503
4	Tiglic Acid	0.2000	0.1000	0.3000	0.1000	0.2000	0.1000
5	Catechin	0.1333	0.0729	0.1667	0.0654	0.1500	0.0779
6	Scopoletin	0.1658	0.0881	0.2434	0.0806	0.1833	0.0813
7	Odoratone	0.0725	0.0319	0.0937	0.0454	0.0467	0.0358
8	Tirucalol	0.0580	0.0524	0.1396	0.0442	0.0867	0.0597
9	Nimbin	0.0632	0.0381	0.0861	0.0285	0.0533	0.0353
10	Nimbolide	0.0748	0.0540	0.0914	0.0322	0.0933	0.0418
11	Sugiol	0.1284	0.0703	0.1937	0.0638	0.1267	0.0672

The utility and regret measures and the corresponding VIKOR rankings were calculated, as mentioned in the previous sections. The finalized decisions on ranking are listed in Table 10.

**TABLE 10:** VIKOR ranking of bioactive Azadrichta indica compounds.

S. No	Compound	$S_{i}$	$R_{i}$	$\Omega_{m}$	Rank
1	Azadirone	0.4147	0.12027	0.40782	5
2	Azadirachtin	0	0	0	1
3	Stigmasterol	0.4773	0.1316	0.45812	7
4	Tiglic Acid	1	0.3	1	11
5	Catechin	0.6662	0.16668	0.61089	8
6	Scopoletin	0.8425	0.24340	0.82695	10
7	Odoratone	0.326	0.09370	0.31917	3
8	Tirucalol	0.4404	0.13958	0.45284	6
9	Nimbin	0.3045	0.08608	0.29573	2
10	Nimbolide	0.3876	0.09333	0.34933	4
11	Sugiol	0.65	0.19367	0.64779	9

### 3.4 SAW ranking of neem chemicals

The matrix calculated for the alternates and criteria was normalized to reduce the dominance of data with higher numerals. This process was achieved using the second phase of the SAW ranking. The resulting normalized matrix is listed in Table 11.

**TABLE 11:** Normalised matrix of bioactive *Azadirachta indica* compounds for SAW ranking.

S. No	Compound	ABC	AUZ	RLI	SLI	MDI	ISLI
1	Azadirone	0.6357	0.5562	0.6141	0.6440	0.6471	0.6004
2	Azadirachtin	1.0000	1.0000	1.0000	1.0000	1.0000	1.0000
3	Stigmasterol	0.5791	0.4999	0.5775	0.5065	0.5882	0.5649
4	Tiglic Acid	0.1108	0.0692	0.0375	0.0798	0.1176	0.1345
5	Catechin	0.4073	0.3215	0.4652	0.3986	0.3382	0.3256
6	Scopoletin	0.2629	0.1800	0.2191	0.2579	0.1912	0.2967
7	Odoratone	0.6775	0.7033	0.6994	0.5820	0.7941	0.6904
8	Tirucalol	0.7423	0.5126	0.5521	0.5935	0.6176	0.4837
9	Nimbin	0.7190	0.6457	0.7238	0.7374	0.7647	0.6947
10	Nimbolide	0.6673	0.4977	0.7067	0.7038	0.5882	0.6380
11	Sugiol	0.4291	0.3459	0.3786	0.4131	0.4412	0.4183

The data in the normalized matrix are aggregated as mentioned in the phase narration. From the obtained aggregation, the SAW ranking was assigned, as shown in Table 12.

**TABLE 12:** SAW ranking of bioactive *Azadirachta indica* compounds.

S. No	Compound	ABC	AUZ	RLI	SLI	MDI	ISLI	Summation	Rank
1	Azadirone	0.1271	0.0556	0.1842	0.0644	0.1294	0.0600	0.6209	5
2	Azadirachtin	0.2000	0.1000	0.3000	0.1000	0.2000	0.1000	1.0000	1
3	Stigmasterol	0.1158	0.0500	0.1733	0.0506	0.1176	0.0565	0.5638	7
4	Tiglic Acid	0.0222	0.0069	0.0112	0.0080	0.0235	0.0134	0.0853	11
5	Catechin	0.0815	0.0322	0.1396	0.0399	0.0676	0.0326	0.3932	9
6	Scopoletin	0.0526	0.0180	0.0657	0.0258	0.0382	0.0297	0.2300	10
7	Odoratone	0.1355	0.0703	0.2098	0.0582	0.1588	0.0690	0.7017	3
8	Tirucalol	0.1485	0.0513	0.1656	0.0594	0.1235	0.0484	0.5966	6
9	Nimbin	0.1438	0.0646	0.2171	0.0737	0.1529	0.0695	0.7216	2
10	Nimbolide	0.1335	0.0498	0.2120	0.0704	0.1176	0.0638	0.6471	4
11	Sugiol	0.0858	0.0346	0.1136	0.0413	0.0882	0.0418	0.4054	8

**FIGURE 5:**
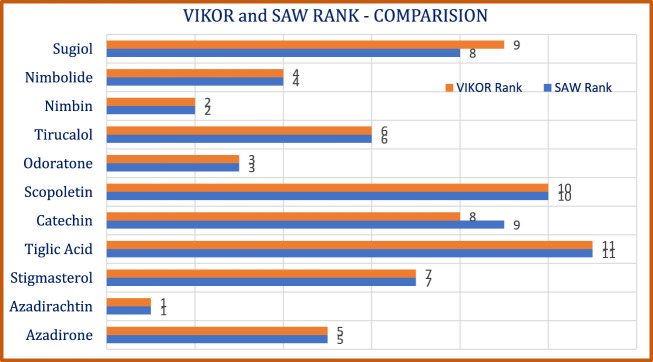
Ranking comparison of Bioactive *Azadirachta indica* Compounds -VIKOR and SAW.

A comparative graphical depiction of the hierarchical arrangement obtained from different techniques is shown in Figure 5.

## 4 Discussion

This research incorporated a range of parameters, such as correlation coefficients between indices and properties, to extract insights from both linear and quadratic regression analyses along with MCDM methodologies. These insights are instrumental in establishing mathematical models that enable the prediction of pharmaceutical compound behavior and hierarchical ranking.

The potential outcomes of the study were evaluated to underpin further investigations aimed at the development of effective neem-based drug formulations. From the statistical metrics of the linear and quadratic regression analyses and MCDM, it can be concluded that.

a) Predictive ability based on reproducibility of the TIs can be ordered as

$$\left(AUZ=SLI=ISLI\right)<\left(MDI=ABC\right)<RLI.$$

b) Prediction accuracy of the properties considered in the study can be ordered as

BP<EV<MR≤Polarization<MM.

c) A nuanced distinction in drug performance was evident, as exemplified by the contrasting rankings of catechin and sugiol. The association between VIKOR and SAW rankings, quantified using the Spearman rank correlation coefficient $\rho =1-\frac{6\sum_{i}{\delta_{i}}^{2}}{N\left(N^{2}-1\right)}$ is 0.98, where $\delta_{i}$ represents the difference between compound rankings.

Regression analysis demonstrated predictive accuracies for various molecular properties, potentially accelerating the identification of promising neem-derived compounds for further development. The compound rankings derived from VIKOR and SAW techniques lend substantial credence and a notable degree of congruence, underscoring the reliability of the proposed ranking system.

To address biodiversity challenges, researchers can use these results to guide the creation of synthetic compounds that replicate the active components of neem. This approach enables drug development in regions in which neem are not readily available. Furthermore, these indices can serve as a bridge between scientists in neem-rich areas and those in countries with limited plant resources, thereby fostering worldwide collaborations in drug discovery. Given the limited global availability of neem, the use of regression models to make analogous predictions could aid pharmacists in creating synthetic compounds that replicate the properties of neem chemicals. The potential outcomes of this study could greatly enhance the clinical application of neem-derived compounds. Drug repurposing for new therapeutic applications, exploration of combination therapies for synergistic effects, and natural product-inspired drug design.

The QSPR model and compound ranking contribute to a) forecasting enzyme inhibition data that align with ligand physicochemical properties, b) elucidating supplementary information extracted from three-dimensional structures, c) minimizing the number of compounds required for synthesis, d) anticipating properties of structurally similar molecules, and e) ascertaining drug composition. Despite these valuable contributions, the development of QSPR models has limitations.

In QSPR, the formulation of optimal regression models is frequently challenged by issues such as overfitting and a lack of three-dimensional data. These challenges restrict the generalization ability of the models, particularly in scenarios involving multiple mechanisms. Overcoming these obstacles could facilitate the development of more robust QSPR models, which are vital for developing models that leverage extensive datasets. Neem is a plant containing over 300 phytochemicals, each exhibiting diverse biological activities, including insecticidal, antimicrobial, and medicinal properties. When ranking and QSPR models were integrated with machine learning techniques, it was possible to evaluate all neem phytochemicals. This integration allows pharmacists to explore their potential for disease treatment, optimize drug formulations, conduct virtual screenings, and perform ADMET profiling.

Chronic illnesses, including cardiovascular and neurological disorders, ischemic conditions, diabetes mellitus, renal impairment, skeletal muscle diseases, and certain cancers, continue to pose significant challenges in the medical field. However, despite these advancements, these conditions often result in drug resistance and adverse outcomes. The integration of experimental research on the bioactive compounds of neem with computational modeling can substantially advance the development of therapeutic strategies for chronic disorders.

## 5 Conclusion

In this study, the properties of neem phytochemicals were evaluated using regression analysis and multi-criteria decision-making methodologies. In regression models, RLI demonstrated the highest reproducibility with 0.83912 and 0.97952 for BP and MM, respectively, with significant p-values, indicating strong correlation with the desired metrics and proves effective in QSPR analysis. Hierarchical rankings demonstrated high concordance, with azadirachtin securing the first rank by providing positive ideal solutions of 40.875, 542.53, 44.4979, 99.675, 68, and 35.887, indicating its highest therapeutic potential. These results aid in rapid compound library assessment and guide the selectivity refinement of neem compounds. The results can be utilized to overcome geographical limitations, facilitate drug optimization, improve predictive accuracy, and enhance virtual screening, bridging drug development and clinical implementation while reducing effort, time, and resources.

## Data Availability

The original contributions presented in the study are included in the article/Supplementary Material, further inquiries can be directed to the corresponding author.
