# Supplementary material for: Predictive modelling and ranking: Azadirachta indica compounds through indices and multi-criteria decision-making techniques
Source: Front Chem. 2025 Apr 29;13:1580267. doi: 10.3389/fchem.2025.1580267 (PMC12069329; doi:10.3389/fchem.2025.1580267)
Supplement: Supplementary file 1 [file DataSheet1.docx]

**Ranking and QSPR of Azadirachta Indica chemicals through Indices and Multi Criteria Decision Making Techniques**

Anuradha D S^1^ , B. Jaganathan^1*^

^1^Department of Mathematics, Vellore Institute of Technology, Chennai, India

email id: [anu.radha2020@vitstudent.ac.in](mailto:anu.radha2020@vitstudent.ac.in)

**Corresponding author:** [**jaganathan.b@vit.ac.in**](mailto:jaganathan.b@vit.ac.in)

**
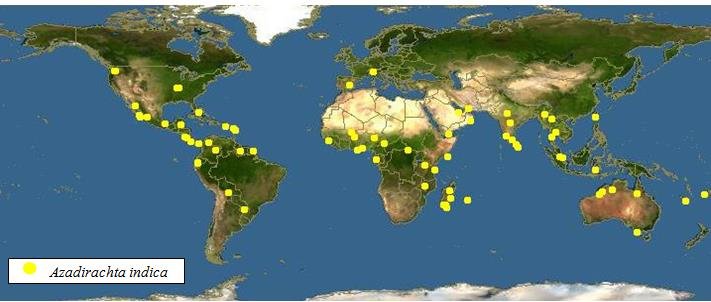
**

Source: <https://www.researchgate.net/publication/326022899/figure/fig1/AS:642312862437379@1530150728953/Map-showing-worldwide-Distribution-of-Azadirachta-indica-in-different-countries-6.png>

**
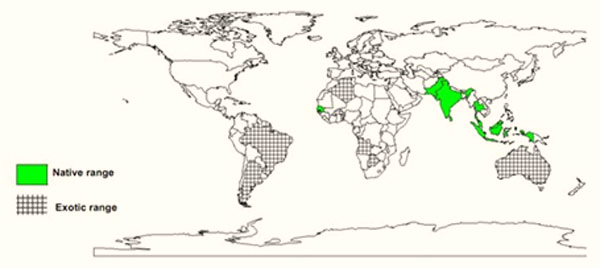
**

**Source:** <https://worldneemorganisation.org/images/worldmap9.jpg>

**Linear Regression Model Equations for 10 indices and five physio-chemical properties:**

**Linear Regression Model for ABC Index:**

$$BP=12.511+234.27[ABC\left( \tau\right)]$$

$$EV=1.9384+39.259[ABC\left( \tau\right)]$$

$$MR=4.1407+11.964[ABC\left( \tau\right)]$$

$$PO=1.641+4.7596[ABC\left( \tau\right)]$$

$$MM=16.477+14.53[ABC\left( \tau\right)]$$

**Linear Regression Model for M_1_ Index:**

$$BP=1.6337+251.26 [MI\left( \tau\right)]$$

$$EV=0.2463+42.985 [MI\left( \tau\right)]$$

$$MR=0.5131+22.197 [MI\left( \tau\right)]$$

$$PO=0.2033+8.821 [MI\left( \tau\right)]$$

$$MM=2.1021+45.187 [MI\left( \tau\right)]$$

**Linear Regression Model for AUZ Index:**

$$BP=0.9103+284.57 [AUZ\left( \tau\right)]$$

$$EV=0.1446+46.082 [AUZ\left( \tau\right)]$$

$$MR=0.2922+30.991 [AUZ\left( \tau\right)]$$

$$PO=0.1158+12.299 [AUZ\left( \tau\right)]$$

$$MM=1.1997+80.547 [AUZ\left( \tau\right)]$$

**Linear Regression Model for RLI :**

$$BP=11.778+239.32[RLI\left( \tau\right)]$$

$$EV=1.6594+44.723 [RLI\left( \tau\right)]$$

$$MR=3.482+23.671 [RLI\left( \tau\right)]$$

$$PO=1.3802+9.3995[RLI\left( \tau\right)]$$

$$MM=14.31+50.214 [RLI\left( \tau\right)]$$

**Linear Regression Model for SLI :**

$$BP=5.3315+238.11 [SLI\left( \tau\right)]$$

$$EV=0.7717+42.956 [SLI\left( \tau\right)]$$

$$MR=1.6216+20.897 [SLI\left( \tau\right)]$$

$$PO=0.6426+8.3029 [SLI\left( \tau\right)]$$

$$MM=6.7562+33.822 [SLI\left( \tau\right)]$$

**Linear Regression Model for ISI :**

$$BP=7.1242+249.33 [ISI\left( \tau\right)]$$

$$EV=10.943+41.882 [ISI\left( \tau\right)]$$

$$MR=2.2708+20.305 [ISI\left( \tau\right)]$$

$$PO=0.8999+8.0673 [ISI\left( \tau\right)]$$

$$MM=9.232+40.182 [ISI\left( \tau\right)]$$

**Linear Regression Model for MLI :**

$$BP=17.67+245.06 [MLI\left( \tau\right)]$$

$$EV=2.8557+38.895 [MLI\left( \tau\right)]$$

$$MR=5.8513+15.484 [MLI\left( \tau\right)]$$

$$PO=2.3185+6.1619 [MLI\left( \tau\right)]$$

$$MM=23.831+19.908 [MLI\left( \tau\right)]$$

**Linear Regression Model for MDI:**

$$BP=6.6714+272.86 [MDI\left( \tau\right)]$$

$$EV=1.0936+42.641 [MDI\left( \tau\right)]$$

$$MR=2.3298+20.15 [MDI\left( \tau\right)]$$

$$PO=0.9233+8.055 [MDI\left( \tau\right)]$$

$$MM=9.2534+47.778 [MDI\left( \tau\right)]$$

**Linear Regression Model for MMRDI:**

$$BP=6.7519+243.11 [MMRDI\left( \tau\right)]$$

$$EV=1.0433+40.751 [MMRDI\left( \tau\right)]$$

$$MR=2.227+15.207 [MMRDI\left( \tau\right)]$$

$$PO=0.8825+6.0478 [MMRDI\left( \tau\right)]$$

$$MM=8.9694+22.969 [MMRDI\left( \tau\right)]$$

**Linear Regression Model for ISLI:**

$$BP=14.866+240.35[ISLI\left( \tau\right)]$$

$$EV=2.4211+37.426[ISLI\left( \tau\right)]$$

$$MR=4.7214+17.771[ISLI\left( \tau\right)]$$

$$PO=1.871+7.0624[ISLI\left( \tau\right)]$$

$$MM=19.695+20.346[ISLI\left( \tau\right)]$$

| Physio- Chemical  Property  ABC | BP | Enthalpy of Vaporization | Molar Refraction | Polarizability | Monoisotopic Mass |
| --- | --- | --- | --- | --- | --- |
| N | 11 | 10 | 11 | 11 | 11 |
| R^2^ | 0.7069 | 0.86544 | 0.95382 | 0.95389 | 0.9627 |
| F- statistics | 21.7124 | 51.45599 | 185.8923 | 186.1926 | 232.84 |
| p | 0.001 | 0.000 | 0.000 | 0.000 | 0.000 |
| $\mathbb{A}$ | 12.511 | 1.9384 | 4.1407 | 1.641 | 16.477 |
| $\mathcal{B}$ | 234.27 | 39.259 | 11.964 | 4.7596 | 14.53 |

**Statistical parameters of ABC Index & Physio chemical Properties**

| Physio- Chemical  Property  **M_1_** | BP | Enthalpy of Vaporization | Molar Refraction | Polarizability | Monoisotopic Mass |
| --- | --- | --- | --- | --- | --- |
| N | 11 | 10 | 11 | 11 | 11 |
| R2 | 0.73979 | 0.854433 | 0.89876 | 0.8985 | 0.96157 |
| F- statistics | 25.5878 | 46.9576 | 79.8955 | 76.6758 | 225.2081 |
| p | 0.000 | 0.000 | 0.000 | 0.000 | 0.000 |
| $\mathbb{A}$ | 1.6337 | 0.2463 | 0.5131 | 0.2033 | 2.1021 |
| $\mathcal{B}$ | 251.26 | 42.985 | 22.197 | 8.821 | 45.187 |

**Statistical parameters of M_1_ Index & Physio chemical Properties**

| Physio-Chemical Property  HUZ | BP | Enthalpy | Molar Refraction | Polarizability | Monoisotopic Mass |
| --- | --- | --- | --- | --- | --- |
| N | 11 | 10 | 11 | 11 | 11 |
| R^2^ | 0.70584 | 0.90973 | 0.89599 | 0.896125 | 0.96269 |
| F- statistics | 21.59613 | 80.6204 | 77.53189 | 77.64226 | 232.230 |
| p | 0.001 | 0.000 | 0.000 | 0.000 | 0.000 |
| $\mathbb{A}$ | 0.9103 | 0.1446 | 0.2922 | 0.1158 | 1.1997 |
| $\mathcal{B}$ | 284.57 | 46.082 | 30.991 | 12.299 | 80.547 |

**Statistical parameters of AUZ Index & Physio chemical Properties**

| Physio-Chemical  Property  RLI | BP | Enthalpy | Molar Refraction | Polarizability | Monoisotopic Mass |
| --- | --- | --- | --- | --- | --- |
| N | 11 | 10 | 11 | 11 | 11 |
| R^2^ | 0.8381 | 0.8803 | 0.9027 | 0.9027 | 0.9714 |
| F- statistics | 46.61 | 58.8576 | 83.5087 | 83.5504 | 306.5394 |
| p | 0.000 | 0.000 | 0.000 | 0.000 | 0.000 |
| $\mathbb{A}$ | 11.778 | 1.6594 | 3.4827 | 1.3802 | 14.31 |
| $\mathcal{B}$ | 239.32 | 44.723 | 23.671 | 9.3995 | 50.214 |

**Statistical parameters of RLI & Physio chemical Properties**

| Physio-Chemical  Property  SLI | BP | Enthalpy | Molar Refraction | Polarizability | Monoisotopic Mass |
| --- | --- | --- | --- | --- | --- |
| N | 11 | 10 | 11 | 11 | 11 |
| R^2^ | 0.773142 | 0.8356 | 0.88099 | 0.8809 | 0.9748 |
| F- statistics | 30.67235 | 40.6713 | 66.6255 | 66.56978 | 348.299 |
| p | 0.000 | 0.000 | 0.000 | 0.000 | 0.000 |
| $\mathbb{A}$ | 5.3315 | 0.7717 | 1.6216 | 0.6426 | 6.7562 |
| $\mathcal{B}$ | 238.11 | 42.956 | 20.897 | 8.3029 | 33.822 |

**Statistical parameters of SLI & Physio chemical Properties**

| Physio- Chemical  Property  ISLI | BP | Enthalpy | Molar Refraction | Polarizability | Monoisotopic Mass |
| --- | --- | --- | --- | --- | --- |
| N | 11 | 10 | 11 | 11 | 11 |
| R^2^ | 0.73948 | 0.88608 | 0.925386 | 0.9254 | 0.97496 |
| F- statistics | 25.5464 | 62.2249 | 111.6208 | 111.5461 | 350.41 |
| p | 0.000 | 0.000 | 0.000 | 0.000 | 0.000 |
| $\mathbb{A}$ | 7.1242 | 10.943 | 2.2708 | 0.8999 | 9.232 |
| $\mathcal{B}$ | 249.33 | 41.882 | 20.305 | 8.0673 | 40.182 |

**Statistical parameters of ISI & Physio chemical Properties**

| Physio- Chemical  Property  MLI | BP | Enthalpy | Molar Refraction | Polarizability | Monoisotopic Mass |
| --- | --- | --- | --- | --- | --- |
| N | 11 | 10 | 11 | 11 | 11 |
| R^2^ | 0.66648 | 0.87679 | 0.9002 | 0.8999 | 0.95185 |
| F- statistics | 17.98488 | 56.9346 | 81.18623 | 80.925 | 177.9 |
| p | 0.002 | 0.000 | 0.000 | 0.000 | 0.000 |
| $\mathbb{A}$ | 17.67 | 2.8557 | 5.8513 | 2.3185 | 23.831 |
| $\mathcal{B}$ | 245.06 | 38.895 | 15.484 | 6.1619 | 19.908 |

**Statistical parameters of MLI & Physio chemical Properties**

| Physio- Chemical  Property  MDI | BP | Enthalpy | Molar Refraction | Polarizability | Monoisotopic Mass |
| --- | --- | --- | --- | --- | --- |
| N | 11 | 10 | 11 | 11 | 11 |
| R^2^ | 0.63228 | 0.84133 | 0.9498 | 0.9498 | 0.955 |
| F- statistics | 15.4752 | 42.4178 | 170.2991 | 170.3065 | 191.137 |
| p | 0.003 | 0.000 | 0.000 | 0.000 | 0.000 |
| $\mathbb{A}$ | 6.6714 | 1.0936 | 2.3298 | 0.9233 | 9.2534 |
| $\mathcal{B}$ | 272.86 | 42.641 | 20.15 | 8.055 | 47.778 |

**Statistical parameters of MDI & Physio chemical Properties**

| Physio-Chemical  Property  MMRDI | BP | Enthalpy | Molar Refraction | Polarizability | Monoisotopic Mass |
| --- | --- | --- | --- | --- | --- |
| N | 11 | 10 | 11 | 11 | 11 |
| R^2^ | 0.6958 | 0.84696 | 0.93239 | 0.9323 | 0.96408 |
| F- statistics | 20.5867 | 44.2742 | 124.1144 | 123.9382 | 241.5851 |
| p | 0.001 | 0.000 | 0.000 | 0.000 | 0.000 |
| $\mathbb{A}$ | 6.7519 | 1.0433 | 2.227 | 0.8825 | 8.9694 |
| $\mathcal{B}$ | 243.11 | 40.751 | 15.207 | 6.0478 | 22.969 |

**Statistical parameters of MMRDI & Physio chemical Properties**

| Physio-Chemical  Property  ISLI | BP | Enthalpy | Molar Refraction | Polarizability | Mono Isotropic Mass |
| --- | --- | --- | --- | --- | --- |
| N | 11 | 10 | 11 | 11 | 11 |
| R^2^ | 0.7007 | 0.9100 | 0.8706 | 0.87059 | 0.965625 |
| F- statistics | 21.07813 | 80.96836 | 60.55866 | 60.5488 | 252.8177 |
| p | 0.018 | 0.000 | 0.000 | 0.000 | 0.000 |
| $\mathbb{A}$ | 14.866 | 2.4211 | 4.7214 | 1.871 | 19.695 |
| $\mathcal{B}$ | 240.35 | 37.426 | 17.771 | 7.0624 | 20.346 |

**Statistical parameters of ISLI & Physio chemical Properties**
